# Clinical Relevance of CD4 Cytotoxic T Cells in High-Risk Neuroblastoma

**DOI:** 10.3389/fimmu.2021.650427

**Published:** 2021-04-22

**Authors:** Xao X. Tang, Hiroyuki Shimada, Naohiko Ikegaki

**Affiliations:** ^1^ Department of Anatomy and Cell Biology, College of Medicine, University of Illinois at Chicago, Chicago, IL, United States; ^2^ Departments of Pathology and Pediatrics, School of Medicine, Stanford University, Stanford, CA, United States

**Keywords:** HLA-E, NKG2E/KLRC3, CRTAM, CADM1, TME (tumor microenvironment), neuroblastoma

## Abstract

Neuroblastoma is the most common extracranial childhood solid tumor. The majority of high-risk neuroblastoma is resistant/refractory to the current high intensity therapy, and the survival of these patients remains poor for the last three decades. To effectively treat these extremely unfavorable neuroblastomas, innovative immunotherapy approaches would be the most promising. In this article, we discuss the identity of tumor-infiltrating effector cells and immunosuppressive cells in high-risk neuroblastoma. Neuroblastoma is unique in that it expresses little or no classical HLA Class I and II. In contrast, high-risk neuroblastomas express the stress-responsive non-classical Class I, HLA-E molecule. HLA-E is the ligand of activating receptors NKG2C/E that are expressed on memory NK cells, CD8+T cells and CD4 CTLs. By examining a comprehensive RNA-seq gene expression dataset, we detected relatively high levels of *CD4* expression in high-risk neuroblastoma tissues. The majority of CD4+ cells were CD3+, and thus they were likely tumor-associated CD4+T cells. In addition, high-level of both CD4 and NKG2C/E expression was associated with prolonged survival of the high-risk neuroblastoma patients, but CD8 levels were not, further suggesting that the CD4+ NKG2C/E+ T cells or CD4 CTL conferred cytotoxicity against the neuroblastoma cells. However, this T cell mediated- “protective effect” declined over time, in part due to the progressive formation of immunosuppressive tumor microenvironment. These observations suggest that to improve survival of high-risk neuroblastoma patients, it is essential to gain insights into how to enhance CD4 CTL cytotoxicity and control the immunosuppressive tumor microenvironment during the course of the disease.

## Introduction

Neuroblastoma is the most common extracranial childhood solid tumor and is known for its biological and clinical heterogeneity. There are two distinct biological types of neuroblastoma: favorable neuroblastoma (known by tumor spontaneous regression or curable simply by surgery) and unfavorable neuroblastoma that are deadly malignant tumors. Based on established prognostic factors, including age, disease stage, *MYCN* amplification status, ploidy, segmental chromosomal aberrations at 1p and 11q, and international neuroblastoma pathology classification (INPC), neuroblastoma is divided into three risk-based categories: low-, intermediate- and high-risk groups (1). Among those prognostic factors, INPC is the strongest prognostic factor that categorizes the tumors into Favorable Histology (FH) and Unfavorable Histology (UH) groups (2). Survival of UH neuroblastoma is essentially the same as that of high-risk neuroblastoma (3, 4). The majority (>50%) of high-risk/UH neuroblastoma will resist or refract the current high intensity multimodal therapy (3–6).

Recently, we have described an immunohistochemistry-based sub-classification of the UH neuroblastoma, which subcategorizes them into UH and Extremely Unfavorable Histology (EUH) subsets (7). As shown in **Figures S1A, B**, the EUH subset includes (i) MYC-driven neuroblastoma expressing high MYC and/or MYCN protein (8), (ii) Neuroblastoma with TERT overexpression due to genomic rearrangements (9–11), and (iii) Neuroblastoma of the ALT group due to ATRX loss (12, 13). The remaining tumors of the UH is called the Null group, which does not have the above three characteristics of the EUH tumors (7). The MYC-driven neuroblastoma group includes both *MYCN*-amplified and non-amplified tumors with high MYCN and MYC expression, respectively, and they are among the worst tumors that ultimately kill the patients. Collectively, the EUH neuroblastoma represents the most malignant and chemotherapy-resistant/refractory disease. Within the scope of this article, we discuss the identity of tumor-infiltrating effector cells and immunosuppressive cells in high-risk neuroblastoma tumor tissues. In addition, we propose an antibody-based approach that can maximize the effector’s killing activity against the tumor cells.

As described below, our data suggest that CD4 CTLs are important effector cells against high-risk neuroblastoma. CD4 CTLs can develop from T_H_0 (14, 15), T_H_1, T_H_2 (16), T_H_17 (17), and Treg (18) subsets. However, CD4 CTLs derived from T_H_1 cells represent the majority of CD4 CTLs (19). A hallmark of CD4 CTLs is the expression of the transcription factor EOMES (19). Previous studies have pointed that at least in some cases, CD4 CTLs are better effectors than CD8 CTLs (20–22). CD4 CTLs kill target cells by two cytotoxic effector mechanisms (23). The first involves the death receptor/ligand pathway. In that, the effector cells express the death ligands (e.g., FASLG), which bind to their cognate receptors (e.g., FAS) on the target cells to induce apoptotic cell death (24–26). The second cytotoxic mechanism is the directed exocytosis of Granzymes and Perforin into target cells to induce apoptosis (27). Others have suggested that CD4+ T cells can reject MHC Class II negative tumor cells through interplay with other infiltrating macrophages (28) and NK cells (20). In addition, it has been shown that CD4 CTLs can target tumor cells in two different ways: an MHC-restricted fashion (21, 22) and MHC-independent manner (29). Nonetheless, it is not clear how CD4 CTLs can directly engage target cells at the molecular levels when MHC Class II restriction is not applied or the tumor lacks HLA Class II expression.

## Immunophenotype of Neuroblastoma

Neuroblastoma is a neural crest-derived tumor, which lacks classical HLA Class I and II expression (30, 31) and exhibits low mutation rates (32, 33). These characteristics help neuroblastoma cells evade CD8+ T cell-mediated immune surveillance. In contrast, there have been conflicting data on PD-L1 expression in neuroblastoma. Aoki et al. first showed that neuroblastoma was PD-L1 negative (34). Later, others suggested that neuroblastoma cells *per se* expressed PD-L1 (35–38). Recently, Shirinbak et al. reported that PD-L1 protein was expressed predominately on tumor-associated macrophages (TAMs) infiltrating into neuroblastoma tissues at diagnosis and a very few neuroblastoma cells became PD-L1 positive after chemotherapy (39). PD-L1 is an important immune checkpoint molecule; we thus carefully examined this issue by multiplex immunohistochemistry assay. As shown in **Figure S1C**, neither FH nor UH neuroblastoma cells expressed PD-L1, and PD-L1 positive staining was in fact detected on the cell membrane of stromal TAMs (see *Discussion*).

## The Study Cohort

**Figure 1:**
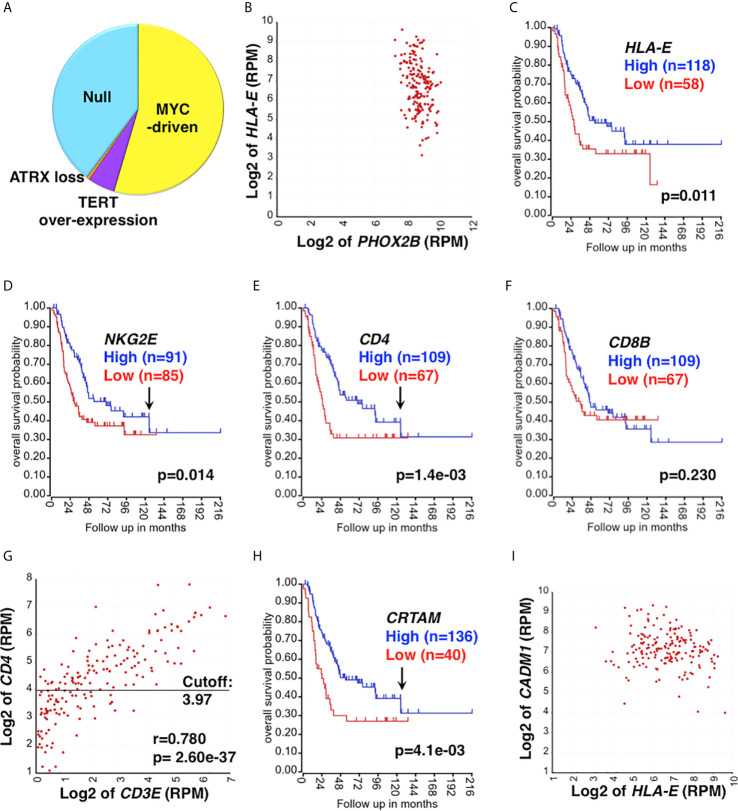
Clinical relevance of CD4 CTL in high-risk neuroblastoma. **(A)** The study cohort and its UH subgroups. The study cohort is composed of 176 high-risk neuroblastoma specimens (40, 41), which include the Null group and EUH groups (MYC-driven, TERT over-expression, and ATRX loss). The proportion of each subset was estimated based on expression levels of *MYCN*, *MYC*, *TERT* and *ATRX*. **(B)** *HLA-E* is highly expressed in high-risk neuroblastoma. *HLA-E* expression was correlated with *PHOX2B* expression (a marker of neuroblastoma) in the study cohort. All tumors were *PHOX2B* positive and co-expressed *HLA-E*. **(C)** The effect of *HLA-E* expression on survival of high-risk neuroblastoma. High *HLA-E* expression was associated with better outcome of high-risk neuroblastoma. Survival of high-risk neuroblastoma patients with high or low expression of *HLA-E* was analyzed by the R2: Genomics Analysis and Visualization Platform (http://r2.amc.nl). **(D–F)** The effect of *NKG2E*, *CD4* and *CD8* expression on survival of high-risk neuroblastoma. **(D)** High *NKG2E* and **(E)** high *CD4* expressions were associated with prolonged survival of high-risk neuroblastoma. Difference in High expression (Blue) vs. Low expression (Red) was statistically significant up to 130 months after diagnosis as indicated by the arrows in **(D, E)**. **(F)** No association between *CD8* expression and disease outcome was found. *CD8* expression is represented by CD8 β chain (*CD8B*). **(G)** Correlation between *CD4* and *CD3E* expressions in the high-risk neuroblastoma. The expression of *CD4* and *CD3E* were highly correlated each other, suggesting the presence of tumor-infiltrating CD4+ T cells. *CD3E* (encoding the CD3ε chain) expression represents CD3 expression. The horizontal bar represents the cutoff value to separate the cohort into high and low *CD4* subsets, which were used in the survival analysis shown in **(E)**. **(H)** The effect of *CRTAM* expression on high-risk neuroblastoma. High *CRTAM* expression, encoding a CTL activating receptor, was associated with prolonged survival of high-risk neuroblastoma. **(I)** High-risk neuroblastoma expresses both *HLA-E* and *CADM1*. All the high-risk tumors examined expressed both *HLA-E* and *CADM1*. *CADM1*, encoding the CRTAM ligand, expressed at high levels in high-risk neuroblastoma. *CADM1* expression also showed a trend of being associated with better survival (p=0.096) (not shown). Unit of expression levels is expressed as Reads Per Million (RPM). Expression levels of genes shown in the figures were expressed as log2 of RPM.

The majority of high-risk neuroblastoma is resistant to the current multimodal therapy, and this group of the tumors is our particular interest. The study cohort includes 176 high-risk neuroblastoma specimens collected at diagnosis (40, 41), and its subsets are shown in **Figure 1A**.

## Clinical Relevance of *HLA-E* Expression in High-Risk Neuroblastoma

HLA-E is a stress-induced molecule (42, 43), and its expression is significantly associated with high-risk neuroblastoma (Stage 4 and *MYCN* amplified cases) (44). We also found that all high-risk neuroblastoma examined expressed *HLA-E* (**Figure 1B**). These observations suggest that high-risk neuroblastoma are under the environmental and/or oncogenesis-associated stress, which in turn forces neuroblastoma cells to express HLA-E. HLA-E is the ligand of activating receptors NKG2C and NKG2E that are expressed on CD8+T cells, CD4 CTLs, and memory/adaptive NK cells (45–48). HLA-E is also the ligand of the inhibitory receptor NKG2A on CD8 T, CD4 CTLs, late immature and mature NK cells (19, 49). It is known that the NK cells can effectively kill HLA-E+ target cells by the ligation of NKG2C/E and HLA-E, followed by the release of effector molecules (Granzymes, Perforin) (47, 50–53). However, little is known whether CD8 T cells and CD4 CTLs would use the same molecular mechanism to directly engage and ultimately lyse the HLA-E+ target cells. To investigate a possible involvement of HLA-E in the anti-tumor immune response against high-risk neuroblastoma, we first examined survival of the patients based on *HLA-E* expression. As shown in **Figure 1C**, high *HLA-E* expression was significantly associated with longer survival of high-risk neuroblastoma, suggesting HLA-E is a target of the effector cells.

## CD4 CTL as Effector Cells Against High-Risk Neuroblastoma

Because of the limited availability of tumor specimens, it is virtually impossible to perform live cell-based analyses on a large high-risk human neuroblastomas cohort. To gain an insight into the identity of immune effector cells against high-risk neuroblastoma, we analyzed a comprehensive RNA-seq gene expression dataset of neuroblastoma (40, 41), using the R2 Genomics Analysis Platform (http://r2.amc.nl).

Neuroblastoma tissues contain the majority of neuroblastoma cells and various stromal cells, including lymphoid and myeloid cells, endothelial cells, Schwann cells and fibroblasts. RNA-seq analysis can detect low-level transcripts from the stroma and tumor-infiltrating immune cells. As shown in **Figure 1D**, we detected expression of the activating receptor *NKG2E* in high-risk neuroblastoma, and high *NKG2E* expression was significantly associated with prolonged survival of high-risk neuroblastoma patients, suggesting the presence of tumor-infiltrating NKG2E+ effector cells in the high-risk neuroblastoma tissues. We also observed that high *CD4* expression (**Figure 1E**), but not *CD8* expression (**Figure 1F**), was associated with better outcome of the patients. Correlation analysis indicated that *CD4* and *CD3E* expressions were significantly associated with each other in the tumor tissues (**Figure 1G**), suggesting the presence of tumor-infiltrating CD4+ T cells. Of note, there was a population of *CD3E* ^negative-low^ and *CD4* ^low^ cases (**Figure 1G**), which could represent non-T CD4+ cells. Even when these cases are excluded from the analysis, association of survival with *CD4* expression still holds. Specifically, excluding 12.5% and 25% of *CD3E* ^negative-low^ cases from the survival analysis gives rise to p values of 0.021 and 0.03, respectively.

**Figure 2:**
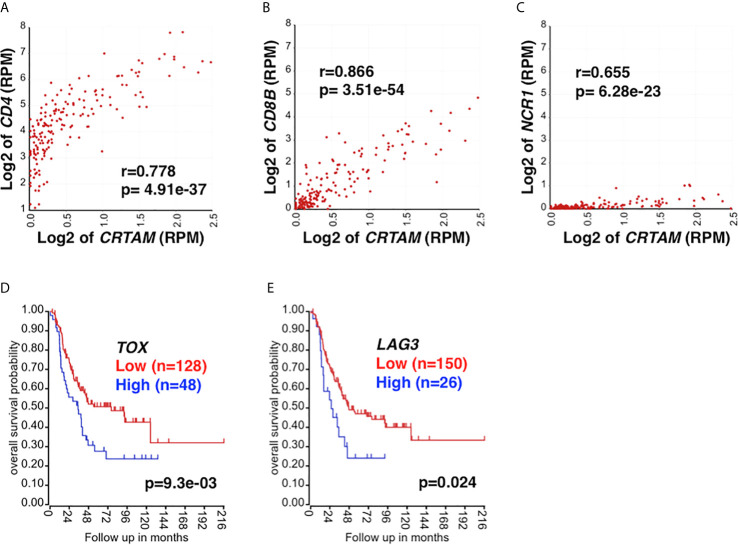
The relationship between *CRTAM* expression and CTL marker expression. CRTAM is an activating receptor of cytotoxic lymphoid cells: CD4 CTLs, CD8 T cells, and NK cells (19, 55) and therefore represents a collective marker of CTLs. **(A)** *CRTAM* and *CD4* expressions were highly correlated each other in high-risk neuroblastoma tissues, suggesting the presence of CRTAM+ CD4+ T cells. **(B)** The expressions of *CD8B* (a CD8 T cell signature) and **(C)** *NCR1* (an NK cell signature) were also found correlated with that of *CRTAM*, although the expression levels of these genes were much lower than that of *CD4*. Taken together, the results suggest that CD4 CTLs are the main CTL subset in high-risk neuroblastoma. CTL exhaustion and the dysfunctional immune response against high-risk neuroblastoma. High-level expression of T-cell exhaustion markers, *TOX* **(D)** and *LAG3* **(E)** was associated with rapid progression and worse outcome of high-risk neuroblastoma, suggesting T-cell exhaustion had occurred in the high-risk neuroblastoma. Blue: High *TOX/LAG3* expression, Red: Low *TOX/LAG3* expression. Unit of expression levels is expressed as Reads Per Million (RPM). Expression levels of genes shown in the figures were expressed as log2 of RPM.

Similar to *NKG2E*, high-level expression of *NKG2C* showed a trend (p=0.09) toward being associated with prolonged survival of high-risk neuroblastoma patients (data not shown). Furthermore, high *CRTAM* expression was associated with better outcome of the patients (**Figure 1H**). CRTAM is known as an activating receptor expressed on CD4 CTLs, CD8 T cells, and NK cells and therefore a collective marker of CTLs (54, 55). On the other hand, neuroblastoma cells expressed CADM1, the ligand of CRTAM (56) (**Figure 1I**). The results shown in **Figure 2A** further suggested the presence of CRTAM+CD4+ cells in high-risk neuroblastoma tissues, which were more abundant than CRTAM+ CD8+ cells (**Figure 2B**) and CRTAM+ NCR1+ cells (i.e, NK cells) (**Figure 2C**). Together, the data suggest that the effector cells of high-risk neuroblastoma are CD4+ CD3+ NKG2C/E+ CRTAM+, namely CD4 CTLs.

Of note, the protective effect of high *NKG2E* expression, high *CD4* expression and high *CRTAM* expression declined over time with a similar kinetics (~130 months after diagnosis) as indicated by the arrows in **Figures 1D, E, H**. This observation suggests the progressive development of the immunosuppressive tumor microenvironment (TME) during the course of high-risk disease.

## A Proposed Model for How CD4 CTLs Engage Neuroblastoma Cells Lacking HLA Class II

Based on our observations, we hypothesize that CD4 CTLs use two additive signals to directly engage neuroblastoma cells lacking HLA Class II: first, ligation between the activating receptors NKG2C/E on CD4 CTLs and the ligand HLA-E on neuroblastoma cells, and second, CRTAM of CD4 CTLs and CADM1 on neuroblastoma cells. Thus, CD4 CTLs act like NK cells and their effector mechanism would be TCR- and HLA Class II-independent. Our observation that almost all the high-risk neuroblastomas examined highly expressed both *HLA-E* and *CADM1* lends support for this hypothesis (**Figure 1I**). In addition, a previous report suggests that CADM1 is a candidate of tumor suppressors for neuroblastoma at the chromosome 11q23 (56), and the patients with tumors having lost CADM1 expression on cell surface have poor prognosis (56). These observations are consistent with our hypothesis that CRTAM is an important receptor on the effector cell against high-risk neuroblastoma.

To further address the hypothesis, we examined the likely cytotoxic pathway involved in the tumor killing of high-risk neuroblastoma. We first found that *FAS* expression was not associated with survival of high-risk neuroblastoma patients (**Figure S2A**). This was in part due to the low expression of *FAS* on the tumor cells compared to *HLA-E* (**Figures S2B, C**). Thus, it is unlikely that CD4 CTL use the FASL/FAS pathway as an effector mechanism. In contrast, *GZMA*/*GZMB* expression was associated with longer survival of high-risk neuroblastoma patients (p=0.026 for both, not shown). Expression of *OX40*, encoding a co-stimulatory molecule on CD4 CTLs, was also associated with better outcome (**Figures S2D, E**). A similar trend was observed for *4-1BB*, which was expressed at lower levels than *OX40* (**Figures S2F, G**). Together, these observations suggest that the anti-neuroblastoma effect of CD4 CTLs relies on the perforin/granzyme pathway.

## The Immunosuppressive Cells in the TME of High-Risk Neuroblastoma

Tumor-associated macrophages (TAMs) include M1 and M2 TAMs. TAMs tend to polarize toward an M2 state (anti-inflammatory, pro-tumor) in the TME and mediate immune exclusion and suppression, and ultimately promote tumor growth. Myeloid-derived suppressor cells (MDSCs) represent a heterogeneous population of immature myeloid cells that inhibit anti-tumor activities of T and NK cells and stimulate Treg, leading to tumor progression (57). As shown in **Figures S3A, B**, we detected the significantly correlated expression of M2 TAMs marker genes (*CD163*, *CD204*, *CD206*) and M-MDSC marker genes (*CD11B*, *CD14*, *CD33*) in the high-risk neuroblastoma examined, suggesting that the various numbers of M2 TAMs and MDSCs are present in the high-risk neuroblastoma TME.

Based on the gene expression profiling analysis, high-risk neuroblastoma tissues express relatively high levels of *CCL2*, *CXCL12*, and *TGFB1*, which could influence the recruitment and polarization of myeloid cells (58–65). We therefore addressed whether the various quantities of M2 TAMs and M-MDSCs linked to the variation in the cytokine/chemokine levels in the TME. To this end, we found that expressions of M2 TAMs markers (*CD163*, *CD204* and *CD206*) and M-MDSC markers (*CD11B*, *CD14* and *CD33*) were all correlated significantly with the expression of *CCL2, CXCL12* and *TGFB1* (**Figure S3C**). Moreover, the expression levels of *TGFB1*, *CXCL12*, and *CCL2* were also highly correlative to each other in all the tumors specimens (**Figure S3D**). Taken together, these observations suggest that the production of these cytokines and chemokines by neuroblastoma and cells in the TME likely determines the quantity of M2 TAMs and M-MDSCs in high-risk neuroblastoma.

Tregs are a subset of CD4+T cells, which maintain peripheral tolerance and suppress anti-tumor immune responses. Tregs interact with infiltrating lymphocytes, stromal cells and tumor cells to exert their immunosuppressive effects (66). CD4+Treg cells are distinguished from other T_H_ lineages *via* FOXP3 expression. FOXP3 stabilizes the suppressive phenotype and capabilities of Treg. CD4+ FOXP3+ Treg express characteristic receptors including CTLA4, GITR, and CD25 (67). Our analysis showed that *FOXP3* expression correlated with *CTLA4* (r=0.837 p= 2.14e-47) (**Figure S3E**), *GITR* (r=0.769 p= 1.28e-35) and *CD25* (r=0.490 p= 4.92e-12) (data not shown), indicating the presence of Tregs in the TME of high-risk neuroblastoma.

TOX has been recognized in driving the epigenetic enforcement of exhaustion (68, 69). Exhausted T cells also express inhibitory receptors: PD-1, CTLA4, LAG3, and TIM3 (70). As shown in **Figures 2D, E**, high *TOX* and *LAG3* expression was associated with adverse outcome of high-risk neuroblastoma, suggesting the exhaustion of CD4 CTLs. Taken together, our analysis suggests the roles of M2 TAMs, MDSCs, Treg, and T cell exhaustion in high-risk neuroblastoma in promoting tumor progression. The results also suggest that the HLA-E reactive CD4 CTL effector cells are functionally compromised in the TME of high-risk neuroblastoma.

## Discussion

There have been several studies examining the immune cell profile in neuroblastoma tissues (71–73). In these reports, the emphasis was on the relationship between immune cell gene expression signatures and clinical outcomes. An additional study employed the immune-related gene expression signature to subdivide the high-risk group into further subsets (74). In this report, we conducted a series of analyses to determine the identity of specific immune effector and immunosuppressive cells in the high-risk neuroblastoma TME. Furthermore, we were particularly interested in the expression of HLA-E, which was expressed on high-risk neuroblastoma (44) (**Figure 1B**). Therefore, identification of effector cells against HLA-E+ tumors would help advance therapeutic strategy against these most malignant tumors.

This study suggests that CD4 CTLs are important effector cells against high-risk neuroblastoma, but their “protective effect” declines over time in part due to the progressive formation of the immunosuppressive TME, leading to the death of the patients. To improve survival of high-risk neuroblastoma patients, therapeutic strategy should include two essential tasks simultaneously: one to enhance the cytotoxicity of CD4 CTLs, and the other to remove the immunosuppressive TME.

Current understanding on cell surface molecules expressed on CD4 CTLs can provide significant insight into the first task. CD4 CTLs express not only the activating receptors NKG2C/E, but also the inhibitory receptor NKG2A, and these activating and inhibitory receptors NKG2C/E/A share the same ligand HLA-E expressed on neuroblastoma cells. CD4 CTLs also express other co-stimulatory molecules on their surface: CRTAM, 4-1BB and OX40. The biological functions of these CD4 CTLs’ cell surface molecules suggest that activating signals by NKG2C/E, CRTAM, 4-1BB, OX40 and the inhibitory signal by NKG2A determine the overall cytotoxicity of CD4 CTL. Thus, blocking of the inhibitory signal NKG2A by anti-NKG2A antibody would enhance the tumor killing of CD4 CTL. On the other hand, the use of agonistic antibodies against 4-1BB and OX40 would maximize the activating signals. This antibody-based approach *via* agonistic/antagonistic antibodies could in turn augment cytotoxicity of CD4 CTLs and result in a robust anti-neuroblastoma immune response.

Effective immunotherapy against solid tumors depends on how to remove the activity of the immunosuppressive TME. To date, there have been numerous studies describing innovative strategies to inactivate the immunosuppressive TME in adult cancers, and each of these studies has focused on one immunosuppressive cell type: TAMs (75–80) or MDSCs (81–83). Our analysis suggests that multiple immunosuppressive cells exist in the high-risk neuroblastoma tissues (M2 TAMs, MDSCs, Treg, exhausted T cells). In addition, a previous study reported that there were protumorigenic cancer-associated fibroblasts in the TME in neuroblastoma (84). Basic understanding of the biology of high-risk neuroblastoma, their metastatic/biological behavior, and knowledge on metabolism of the immune cells would help advance our strategy toward how to systematically remove the immunosuppressive TME and restore effector functions of the immune cells.

Intriguingly, our data show that neuroblastoma cells do not express PD-L1 (**Figure S1C**). The PD-1/PD-L1 ligation inhibits T-cell receptor signaling in effector T cells. Therefore, the lack of classical HLA Class I expression makes it unnecessary for the neuroblastoma cells to utilize the PD-1/PD-L1 pathway to avoid the killing by PD-1+ CD8 CTLs. On the other hand, we propose that in the absence of CD8+ T-mediated immunity, CD4 CTLs can target the HLA-E+ high-risk neuroblastoma cells in a TCR- and HLA-independent manner, which in turn would be unaffected by the PD-L1 expression status on the neuroblastoma cells.

Because of the biological heterogeneity of high-risk neuroblastoma, multiple immunotherapy protocols would be required to treat the patients. Currently, GD2, a surface glycolipid is the most common target for neuroblastoma immunotherapy. Anti-GD2 monoclonal antibodies have improved event-free survival and overall survival in patients with high-risk neuroblastoma (85, 86). However, some neuroblastomas intrinsically lack GD2 expression (87). Furthermore, in response to anti-GD2 therapy, tumor cells can down-regulate GD2 (88). In this study, we have found that the majority of high-risk neuroblastoma expresses both *HLA-E* and genes responsible for GD2, but ~5% of the tumors were HLA-E+ and likely GD2 negative (**Figures S4A–C**). Combination treatment against HLA-E and GD2 or an alternative therapy to anti-GD2 antibodies would be beneficial to these patients. Therapeutic interventions based on other cell surface molecules on neuroblastoma, including 4Ig-B7-H3 (89) and CD57 (90, 91), might be worth investigating, although these molecules are known to be expressed on both the tumor cells and immune cells.

It should be mentioned that antibody-based approaches targeting tumor surface molecules (e.g., GD2) are mediated by ADCC, which requires immune-active NK cells or macrophages (M1 TAMs). Thus, enhancing the immune-active status of tumor-infiltrating immune cells is a key strategy for anti-neuroblastoma immunotherapy. Lastly, because high-risk neuroblastoma is a metastatic disease, therapeutics given to these pediatric patients should be delivered systemically but tumor-specific with little toxicity to the normal cells. Immunotherapy would be a desirable approach to treat high-risk neuroblastoma patients, as it can target the tumor cells. This is in fact a basic principle underlying how the immune system works.

## Data Availability Statement

Publicly available datasets were analyzed in this study. This data can be found here: NCBI GEO, GSE62564.

## Author Contributions

XT and NI designed study, generated and analyzed the data, and conceptualized, wrote, and edited the manuscript. HS generated and analyzed the IHC data and edited the manuscript. All authors contributed to the article and approved the submitted version.

## Funding

The funding was provided in part by NIH P01CA217959 (Project Leaders Seeger, Maris) to HS and DoD W81XWH-18-1-0418 (PI Asgharzadeh) to HS.

## Conflict of Interest

The authors declare that the research was conducted in the absence of any commercial or financial relationships that could be construed as a potential conflict of interest.

## Supplementary Material

Supplementary Figure 1
**(A)** Neuroblastoma subsets in a general cohort. In a general cohort, about 50% of the tumors are Favorable Histology (FH) group. The remaining 50% tumors belong to the Unfavorable Histology (UH) group, which is further divided into subcategories: the Null group (chemotherapy-sensitive; ~10%) and the Extremely Unfavorable Histology (EUH) group (chemotherapy-resistant/refractory). The EUH tumors include MYC-driven neuroblastomas (high MYCN and/or MYC protein expression; ~30%), TERT overexpression tumors (~7%), and ALT tumors (ATRX loss; ~4%). The proportional distribution of these subsets was estimated based on previous publications from our group (8) and others (9, 10, 92). **(B)** Immunohistochemical phenotypes of UH neuroblastoma subgroups. Examples of immunohistochemistry images of the EUH and Null group neuroblastomas are shown, which was performed as previously described (8, 93, 94). **(C)** Neuroblastoma at diagnosis does not express PD-L1. Multiplex IHC was performed to detect PD-L1 (brown, membrane staining) and PHOX2B (red, nuclear staining, one of the most reliable markers for neuroblastoma cells (95) on FH (5 cases) and UH (5 cases) neuroblastomas at diagnosis. PHOX2B positive neuroblastoma cells were always negative for PD-L1. In contrast, only PHOX2B negative cells, the majority of them appeared to be macrophages, showed positive staining for PD-L1 (see the inset in the UH tumor image). Representative cases of FH and UH IHC images of PD-L1 and PHOX2B staining are shown. The scale bar represents 20μm.

Supplementary Figure 2 Effects of *FAS*, *OX40* and *4-1BB* expressions on survival of high-risk neuroblastoma. **(A)** *FAS* expression was not associated with survival of the high-risk neuroblastoma patients. This was likely due to low-level expression of *FAS* in high-risk neuroblastoma **(B)** as compared to *HLA-E* expression **(C)**. *HLA-E and PHOX2B* expression in **(C)** is the same as **Figure 1B**, and it is shown here for the comparison of *FAS* expression to *HLA-E* expression. *PHOX2B* expression was used as a gene expression control for neuroblastoma. **(D)** High *OX40* expression was significantly associated with better survival of the high-risk patients, and high *4-1BB* expression showed a similar trend **(F)**. The difference in the effects of *OX40* and *4-1BB* expressions on the patient survival was likely due to expression levels of *OX40* and *4-1BB* as shown in **(E, G)**, respectively. *EOMES* expression signifies CD4 CTLs (19, 55, 96), and it was used to assess expression levels of *OX40* and *4-1BB* in tumor-infiltrating CD4 CTLs. **(E)** *OX40* expression among the tumors with *EOMES* expression over 1.0 RPM was significantly higher than that of *4-1BB* **(G)** (p=4.8e-04). Statistical analysis was done using a Student’s t-test. Unit of expression levels is expressed as Reads Per Million (RPM). Expression levels of genes shown in the figures were expressed as log2 of RPM.

Supplementary Figure 3 M2 TAMs, M-MDSCs and Tregs in the TME of high-risk neuroblastoma. **(A)** The expression of M2 TAM signature genes (*CD163, CD204*, and *CD206*) (97, 98) was examined by 3D-correlation analysis in high-risk neuroblastoma. The signature gene expression was tightly associated each other, suggesting that various numbers of M2 TAMs were present in high-risk neuroblastoma tissues. **(B)** The expression of M-MDSC signature genes (*CD11B, CD14* and *CD33*) (99) was also examined by 3D-correlation analysis in high-risk neuroblastoma. Based on histological observations, monocytic MDSCs (M-MDSC) appeared to be the main subset of MDSCs in neuroblastoma. Therefore, only the M-MDSC signature genes were analyzed. As shown, there was a tight association among *CD11B, CD14*, and *CD33* gene expressions in high-risk neuroblastoma tissues, suggesting that various amounts of M-MDSCs were present in the TME. **(C)** Expression of M2 TAM and M-MDSC marker genes was highly correlated with the expression of cytokine and chemokines genes indicated. **(D)** The expression levels of chemokine and cytokine genes (*CCL2*, *CXCL12* and *TGFB1*) are also highly correlated to each other in all tumors examined. These observations suggest that the establishment of M2 TAMs and M-MDSCs in the TME are dependent on the cytokine and chemokines. **(E)** Expressions of Treg signature genes (*FOXP3* and *CTLA4*) in the high-risk neuroblastoma. The expressions of these genes were highly correlated each other, suggesting the presence of Treg cells in the tumor tissues. Unit of expression levels is expressed as Reads Per Million (RPM). Expression levels of genes shown in the figures were expressed as log2 of RPM.

Supplementary Figure 4 The majority of high-risk neuroblastomas co-expressed *HLA-E* and genes responsible for GD2 synthesis (*B4GALNT1* and *ST8SIA1*). GD2 is a glycolipid and an immunotherapy target of neuroblastoma, but its expression cannot directly be measured by gene expression. We thus examined the expression of *B4GALNT1* encoding GD2 synthase and *ST8SIA1* encoding GD3 synthase as surrogates of GD2 expression in high-risk neuroblastoma. Of note, GD3 synthase is the rate-limiting enzyme for GD2 synthesis, and therefore, expression of *ST8SIA1* was included in the analysis **(A, B)**. The result showed that the vast majority of high-risk neuroblastomas co-expressed *HLA-E* together with *B4GALNT1* and *ST8SIA1.* In other words, most tumors are both HLA-E+ GD2+. **(C)** The majority of high-risk neuroblastomas expressed both *ST8SIA1* and *B4GALNT1.* However, about 5% of the tumors expressed very low levels of both *B4GALNT1* and *ST8SIA1* (the red circle), suggesting these tumors (~5%) were negative for GD2 at diagnosis. Expression levels of genes shown in figures were expressed as log2 of RPM. **(D)** Average expression levels of genes examined in the study are shown. Unit of expression levels is expressed as Reads Per Million (RPM). Expression average was calculated based on the high-risk neuroblastoma cases (n=176) (40, 41). *TOX* is expressed in non-T cell lineage cells (e.g., B cells, NK cells, and granulocytes) and its expression levels appear higher than other T cell markers in tumor tissues (https://www.proteinatlas.org/ENSG00000198846-TOX/tissue). Housekeeping genes (HKGs) include *RPL5*, *RPL28*, *TBP*, *RPL13A*, *RPLP0* and *TFRC*.

## Abbreviations

CD4 CTL, CD4 Cytotoxic T Lymphocyte; TCR, T-cell receptor; ADCC, Antibody-dependent cell-mediated cytotoxicity.
