# Supplementary figures and images for: Clinical Relevance of CD4 Cytotoxic T Cells in High-Risk Neuroblastoma
Source: Front Immunol. 2021 Apr 22;12:650427. doi: 10.3389/fimmu.2021.650427 (PMC8101497; doi:10.3389/fimmu.2021.650427)

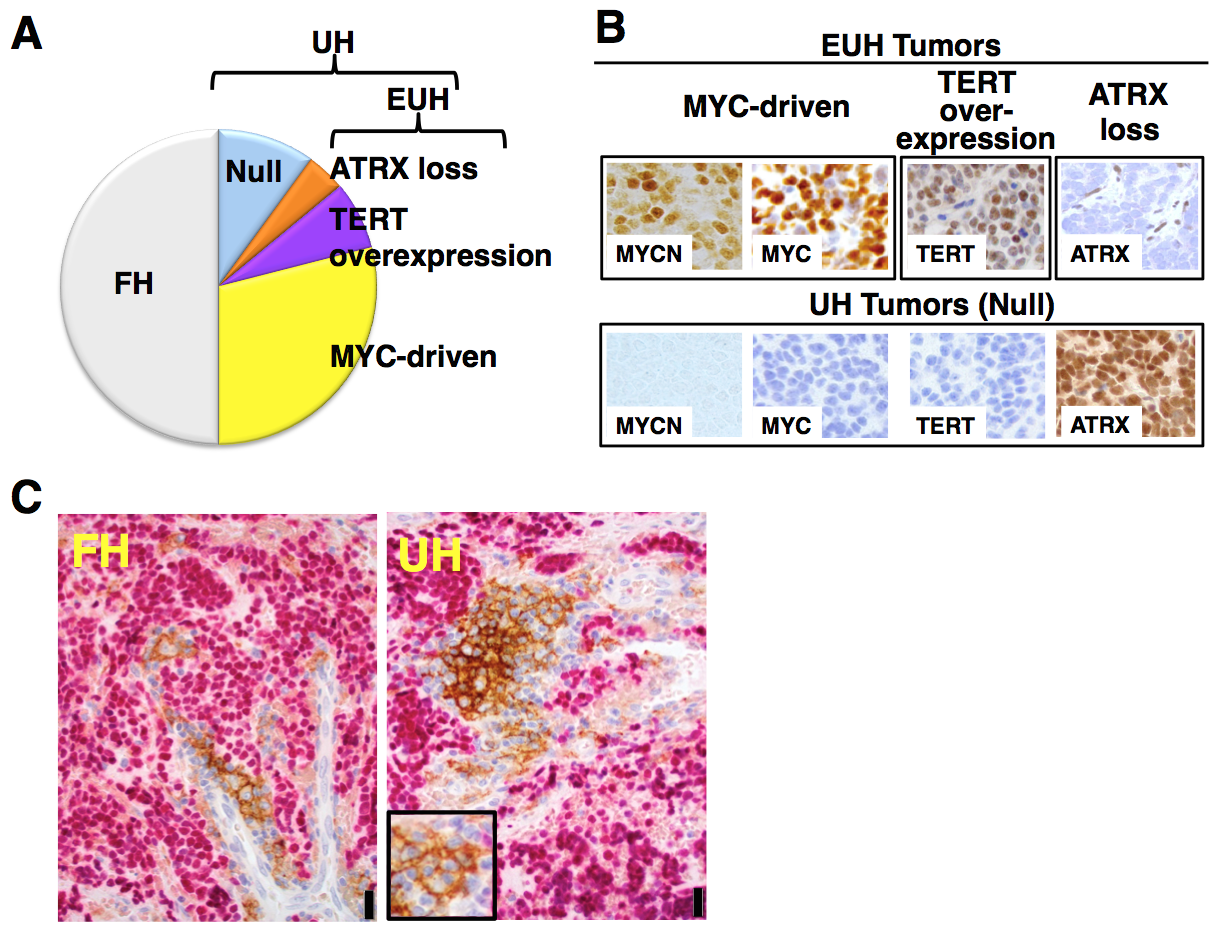

Supplement: Supplementary Figure 1 — (A) Neuroblastoma subsets in a general cohort. In a general cohort, about 50% of the tumors are Favorable Histology (FH) group. The remaining 50% tumors belong to the Unfavorable Histology (UH) group, which is further divided into subcategories: the Null group (chemotherapy-sensitive; ~10%) and the Extremely Unfavorable Histology (EUH) group (chemotherapy-resistant/refractory). The EUH tumors include MYC-driven neuroblastomas (high MYCN and/or MYC protein expression; ~30%), TERT overexpression tumors (~7%), and ALT tumors (ATRX loss; ~4%). The proportional distribution of these subsets was estimated based on previous publications from our group (8) and others (9, 10, 92). (B) Immunohistochemical phenotypes of UH neuroblastoma subgroups. Examples of immunohistochemistry images of the EUH and Null group neuroblastomas are shown, which was performed as previously described (8, 93, 94). (C) Neuroblastoma at diagnosis does not express PD-L1. Multiplex IHC was performed to detect PD-L1 (brown, membrane staining) and PHOX2B (red, nuclear staining, one of the most reliable markers for neuroblastoma cells (95) on FH (5 cases) and UH (5 cases) neuroblastomas at diagnosis. PHOX2B positive neuroblastoma cells were always negative for PD-L1. In contrast, only PHOX2B negative cells, the majority of them appeared to be macrophages, showed positive staining for PD-L1 (see the inset in the UH tumor image). Representative cases of FH and UH IHC images of PD-L1 and PHOX2B staining are shown. The scale bar represents 20μm. [file Image_1.tiff]

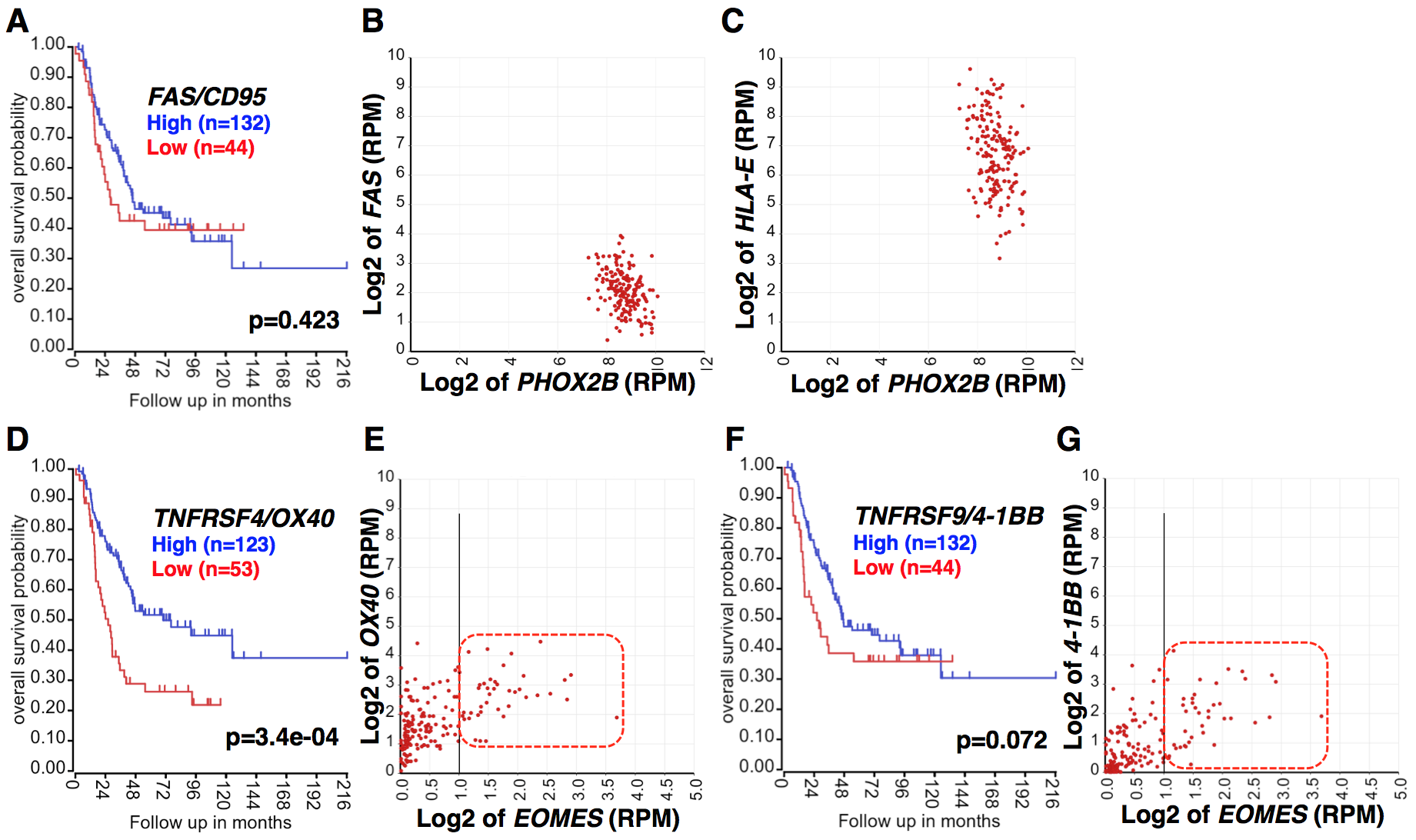

Supplement: Supplementary Figure 2 — Effects of FAS, OX40 and 4-1BB expressions on survival of high-risk neuroblastoma. (A) FAS expression was not associated with survival of the high-risk neuroblastoma patients. This was likely due to low-level expression of FAS in high-risk neuroblastoma (B) as compared to HLA-E expression (C). HLA-E and PHOX2B expression in (C) is the same as Figure 1B , and it is shown here for the comparison of FAS expression to HLA-E expression. PHOX2B expression was used as a gene expression control for neuroblastoma. (D) High OX40 expression was significantly associated with better survival of the high-risk patients, and high 4-1BB expression showed a similar trend (F). The difference in the effects of OX40 and 4-1BB expressions on the patient survival was likely due to expression levels of OX40 and 4-1BB as shown in (E, G), respectively. EOMES expression signifies CD4 CTLs (19, 55, 96), and it was used to assess expression levels of OX40 and 4-1BB in tumor-infiltrating CD4 CTLs. (E) OX40 expression among the tumors with EOMES expression over 1.0 RPM was significantly higher than that of 4-1BB (G) (p=4.8e-04). Statistical analysis was done using a Student’s t-test. Unit of expression levels is expressed as Reads Per Million (RPM). Expression levels of genes shown in the figures were expressed as log2 of RPM. [file Image_2.tiff]

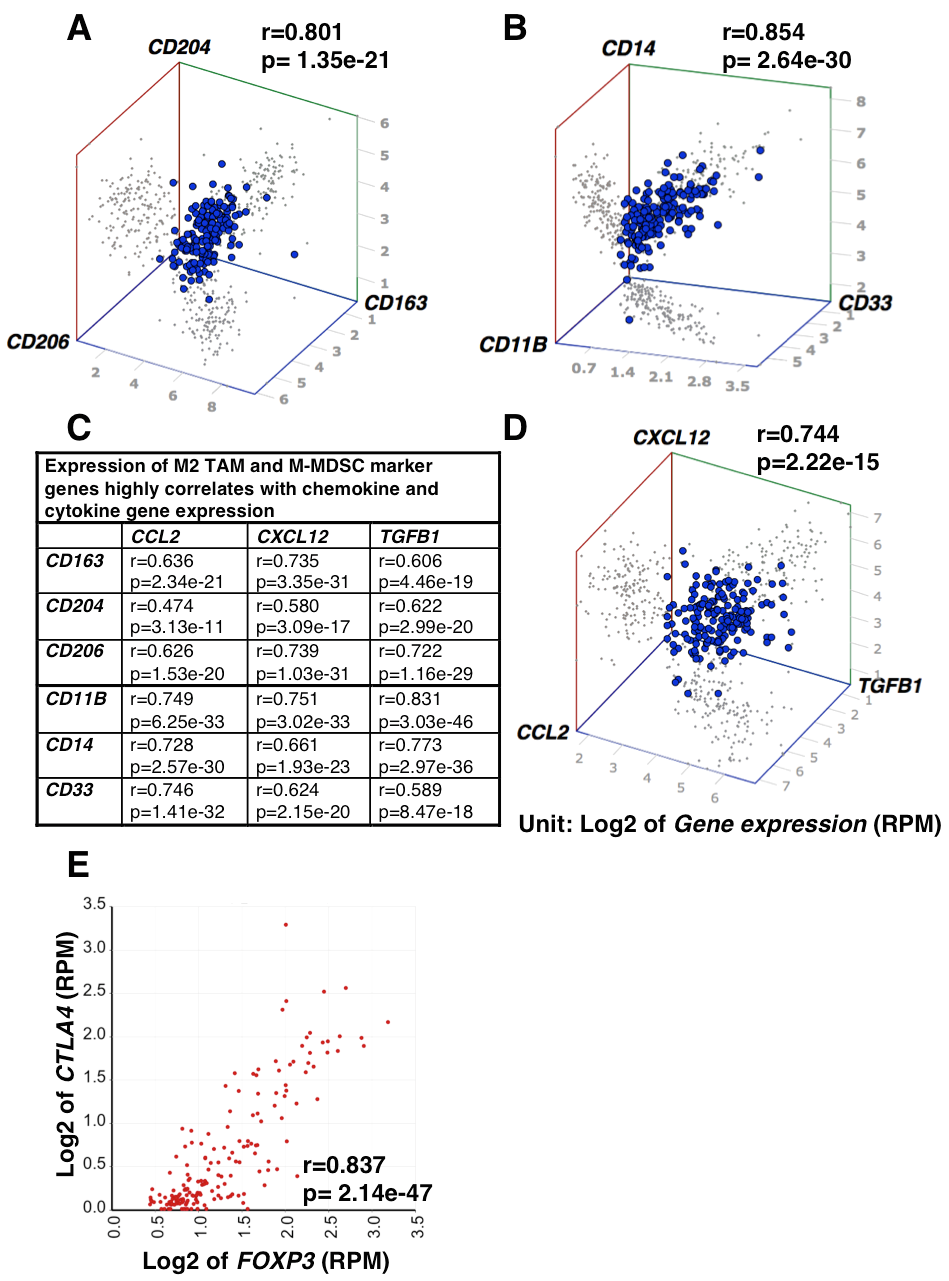

Supplement: Supplementary Figure 3 — M2 TAMs, M-MDSCs and Tregs in the TME of high-risk neuroblastoma. (A) The expression of M2 TAM signature genes (CD163, CD204, and CD206) (97, 98) was examined by 3D-correlation analysis in high-risk neuroblastoma. The signature gene expression was tightly associated each other, suggesting that various numbers of M2 TAMs were present in high-risk neuroblastoma tissues. (B) The expression of M-MDSC signature genes (CD11B, CD14 and CD33) (99) was also examined by 3D-correlation analysis in high-risk neuroblastoma. Based on histological observations, monocytic MDSCs (M-MDSC) appeared to be the main subset of MDSCs in neuroblastoma. Therefore, only the M-MDSC signature genes were analyzed. As shown, there was a tight association among CD11B, CD14, and CD33 gene expressions in high-risk neuroblastoma tissues, suggesting that various amounts of M-MDSCs were present in the TME. (C) Expression of M2 TAM and M-MDSC marker genes was highly correlated with the expression of cytokine and chemokines genes indicated. (D) The expression levels of chemokine and cytokine genes (CCL2, CXCL12 and TGFB1) are also highly correlated to each other in all tumors examined. These observations suggest that the establishment of M2 TAMs and M-MDSCs in the TME are dependent on the cytokine and chemokines. (E) Expressions of Treg signature genes (FOXP3 and CTLA4) in the high-risk neuroblastoma. The expressions of these genes were highly correlated each other, suggesting the presence of Treg cells in the tumor tissues. Unit of expression levels is expressed as Reads Per Million (RPM). Expression levels of genes shown in the figures were expressed as log2 of RPM. [file Image_3.tiff]

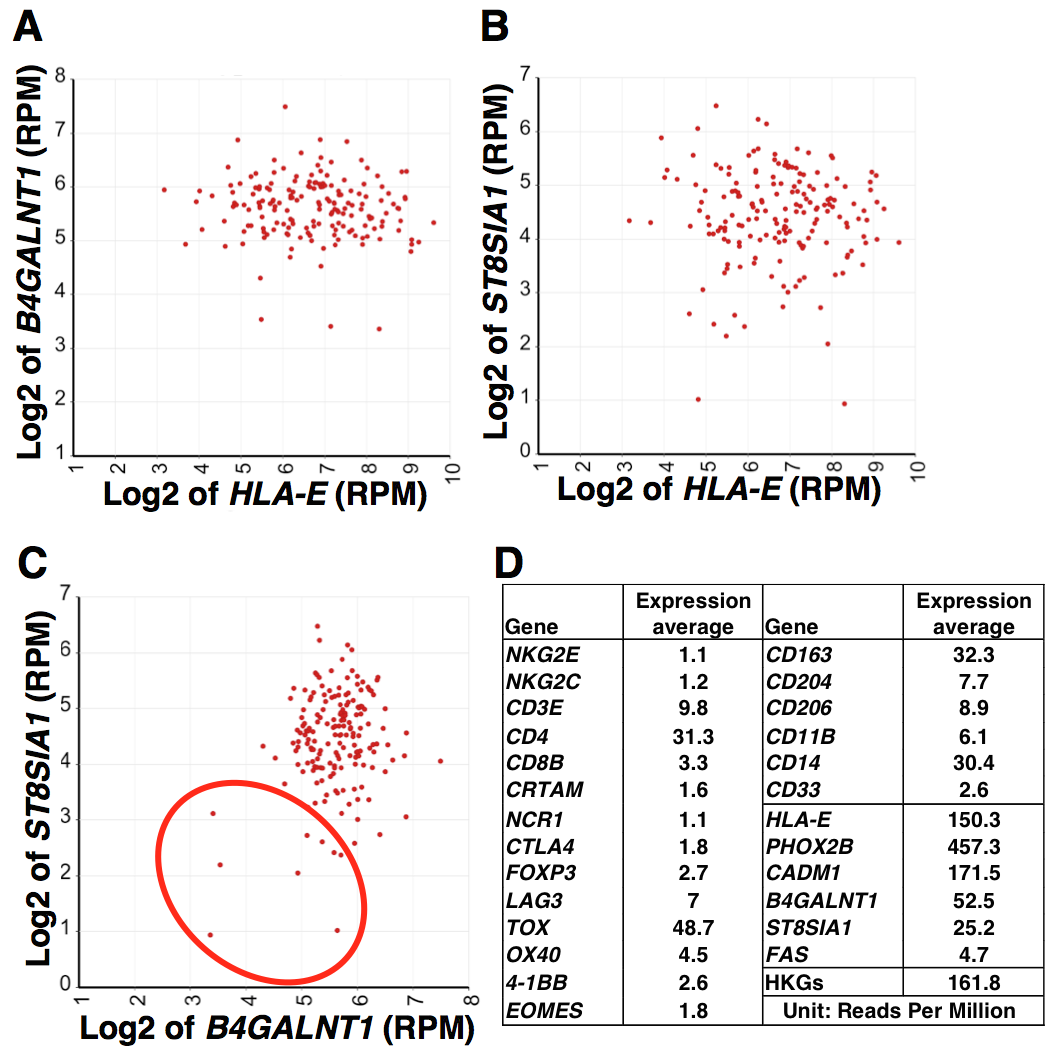

Supplement: Supplementary Figure 4 — The majority of high-risk neuroblastomas co-expressed HLA-E and genes responsible for GD2 synthesis (B4GALNT1 and ST8SIA1). GD2 is a glycolipid and an immunotherapy target of neuroblastoma, but its expression cannot directly be measured by gene expression. We thus examined the expression of B4GALNT1 encoding GD2 synthase and ST8SIA1 encoding GD3 synthase as surrogates of GD2 expression in high-risk neuroblastoma. Of note, GD3 synthase is the rate-limiting enzyme for GD2 synthesis, and therefore, expression of ST8SIA1 was included in the analysis (A, B). The result showed that the vast majority of high-risk neuroblastomas co-expressed HLA-E together with B4GALNT1 and ST8SIA1. In other words, most tumors are both HLA-E+ GD2+. (C) The majority of high-risk neuroblastomas expressed both ST8SIA1 and B4GALNT1. However, about 5% of the tumors expressed very low levels of both B4GALNT1 and ST8SIA1 (the red circle), suggesting these tumors (~5%) were negative for GD2 at diagnosis. Expression levels of genes shown in figures were expressed as log2 of RPM. (D) Average expression levels of genes examined in the study are shown. Unit of expression levels is expressed as Reads Per Million (RPM). Expression average was calculated based on the high-risk neuroblastoma cases (n=176) (40, 41). TOX is expressed in non-T cell lineage cells (e.g., B cells, NK cells, and granulocytes) and its expression levels appear higher than other T cell markers in tumor tissues (https://www.proteinatlas.org/ENSG00000198846-TOX/tissue). Housekeeping genes (HKGs) include RPL5, RPL28, TBP, RPL13A, RPLP0 and TFRC. [file Image_4.tiff]
